# Combination Thyroid Hormone Replacement; Knowns and Unknowns

**DOI:** 10.3389/fendo.2019.00706

**Published:** 2019-10-22

**Authors:** Peter N. Taylor, Vinay Eligar, Ilaria Muller, Anna Scholz, Colin Dayan, Onyebuchi Okosieme

**Affiliations:** Thyroid Research Group, Cardiff University School of Medicine, Systems Immunity Research Institute, Cardiff, United Kingdom

**Keywords:** combination, liothyronine, levothyroxine, hypothyroidism, treatment

## Abstract

Hypothyroidism is common throughout the world and readily diagnosed with thyroid function tests. Management should be straightforward but appears not to be the case. Thyroid hormone replacement with levothyroxine monotherapy is the standard treatment which is effective in the majority of cases. However, 10–15% of patients established on levothyroxine do not feel their health is entirely restored and some patients prefer the addition of liothyronine. Proponents of liothyronine argue that the ratio of T3 and T4 hormones is substantially altered on T4 monotherapy and therefore both hormones may be needed for optimal health. This remains controversial as clinical trials have not demonstrated superiority of combination therapy (levothyroxine and liothyronine) over levothyroxine monotherapy. There is now a pressing need for further studies and in particular randomized controlled trials in this area. To help design and facilitate dedicated trials and better understand thyroid hormone replacement, this review summarizes the evidence where there is established knowledge and agreement (knowns) and areas where research is lacking (unknowns). Agreements include the extent of dissatisfaction with levothyroxine monotherapy, biases in testing for hypothyroidism and prescribing levothyroxine, as well as variable thresholds for prescribing levothyroxine and challenges in liothyronine dosing. The review will also highlight and summarize the unknowns including the long-term safety profile of liothyronine, and potential biomarkers to identify individuals who might benefit most from combination therapy.

## Introduction

Hypothyroidism is common throughout the world and particularly affects females (1). It is readily identifiable and treatable, but if untreated or poorly managed can have profound adverse effects (1, 2). Levothyroxine (LT4) is the current standard treatment although liothyronine (LT3) and desiccated thyroid extract (DTE) are also used (2, 3). Prior to the 1970's both combination thyroid hormone replacement (LT3 + LT4) and DTE were widely prescribed (4). The discovery that T4 is largely activated to T3 outside of the thyroid within target tissues (5) and concerns regarding dosing and stability of FT3 profiles in patients on LT3 or DTE have made LT4 the standard of care for nearly 50 years. Thyroid hormone replacement is now an important global public health issue and as at present, LT4 is the most commonly prescribed medication in the USA and the third most commonly prescribed in the UK (2).

The diagnosis and monitoring of hypothyroidism is largely biochemical and undertaken using easily available laboratory thyroid function tests. The inverse relationship between thyroid hormone and the pituitary derived hormone, thyrotropin (TSH) has lead to TSH becoming the accepted marker of thyroid status (6) and the goal of treatment is therefore to normalize TSH levels (2). Whilst such an approach appears at first instance to be straightforward several issues have been raised in recent years which necessitate further research. These are fundamental and relate to the diagnosis, treatment, and monitoring of thyroid function. As a result, calls have been made that the focus on TSH rather than symptom relief should be re-assessed (7). A joint consideration of TSH, patient symptoms, and a more personalized approach may therefore be required to address the recent surge in patient complaint rates (7). Indeed thyroid hormone, once thought to be the easiest hormone to replace, has become very controversial, with patient groups challenging the assumptions of endocrinologists.

The most pertinent issue is that there are widespread reports of dissatisfaction with LT4 replacement (8) estimated at 10–15% of patients (2, 9). It is difficult to assess the extent to which this reflects dissatisfaction in the general population as undergoing thyroid function testing itself is a predictor of psychological morbidity (10). This effect may be further exaggerated by the dramatic increase in thyroid function testing and initiation of LT4 at lower TSH thresholds in recent years (11, 12).

LT4 monotherapy results in individuals having relatively lower FT3 and higher FT4 levels than individuals with an intact, undisturbed, hypothalamopituitary-thyroid (HPT) axis (13), but the clinical relevance of this finding is controversial (14). Consistently many patients (~20%) may be apparently “over-replaced” with LT4 as evidenced by a low or suppressed TSH (11, 15, 16). There is evidence that a lower TSH can result in greater patient satisfaction (17), but also greater long term risks (18). In the UK in particular, due to widespread concern and media coverage over recent large increases in costs of LT3 and access to treatment (19) more patients are now aware of and desiring combination LT3:LT4 replacement. Increased awareness and self-medication with LT3 has also been observed in Denmark (20) and the USA (8). Four systematic reviews/meta-analyses of trials of combination LT3:LT4 replacement found no clear benefit of combination therapy over LT4 monotherapy (21–24). However, some of the key limitations of these trials (discussed further below) included small sample sizes, variable quality in outcomes assessed, the use of only once daily (“pulsatile”) rather than slow release T3 dosing and the acceptance that feedback to TSH is the same with all forms of thyroid hormone replacement resulting in potential for dose titration errors. Furthermore, there is recent evidence that T4 inhibits the deiodinase that converts T4 to T3 in target tissues, with the potential to result in paradoxically reduced levels of intracellular T3 when there is a high circulating T4/T3 ratio (25).

Based on this uncertainty regarding the benefit of LT3, current European Thyroid Association guidance (26) recognizes that LT4 is the treatment of choice but recommends that a 3-months trial of LT3 could be considered in patients with persistent unexplained symptoms despite good compliance. Likewise, a British Thyroid Association position statement also endorses LT4 as the treatment of choice but acknowledges that a trial of combination therapy could be warranted in patients who have unequivocally failed to respond to LT4 following a discussion with the patient of the uncertain risks and benefits of combination therapy (27). Whilst current American Thyroid Association guidance (28) indicates a trial of LT3 can be considered in carefully selected patients it also highlights no consistent superiority of combination LT3:LT4 therapy. DTE was not endorsed in either the European, British, or American guidelines (26–28).

Set against the uncertain benefits of combined LT3:LT4 therapy a balance has to be made against the potential for adverse consequences of using LT3. The lack of clarity in both these areas results in difficult decisions for patients and clinicians. Equally, maintaining the status quo is not desirable given the dissatisfaction that exists with LT4 monotherapy and the numbers of patients being over-replaced on current monitoring with TSH. A substantial number of patients seek to try LT3 monotherapy or other preparations such as DTE and have sought to source their therapy themselves (19). Failure of endocrinologists to engage sufficiently with these patients may place individuals at unnecessary risk of over-replacement.

Taken together there is a pressing need for further randomized controlled trials in this area, as well as research into longer acting LT3 preparations, better markers which predict tissue thyroid status, better assessments of treatment outcomes, and improved knowledge of the benefit-risk profile of long-term combination LT3:LT4 therapy. A review of the current evidence for combination thyroid hormone replacement with an overview of future research directions is therefore timely. We will summarize and identify areas of existing knowledge and research agreement (the knowns) as well as areas where data are more urgently needed (the unknowns).

## The Knowns

### Serum T4, Not Serum T3 Is the Main Source of T3 Within Cells

In mammals, the majority of active thyroid hormone (T3) within cells is derived not directly from T3 in the circulation, but indirectly from T4, via the action of the D2 deiodinase (29, 30). The concentration of circulating T4 is about five times higher than T3 and it is the circulating T4 rather than T3 that serves as the main source of thyroid hormone for the body (5). In addition a proportion of serum T3 is also derived from serum T4 (5). These considerations have been used as a key theoretical argument against the use of LT3 in the management of hypothyroidism.

However, serum thyroid hormone levels may not entirely reflect intracellular thyroid hormone status. Via variation in uptake, activation and metabolism of T4, cells can modulate intracellular T3 levels independently of circulating thyroid hormone levels. Downstream control by thyroid hormone transporters such as MCT8 and MCT10, the deiodinases (DIO2,DIO3), and transcription co-factors locally modify thyroid hormone bioavailability and action at the intracellular level. The potential for dichotomy between serum thyroid hormone levels and intracellular action is dramatically illustrated in reports of individuals with rare mutations in the MCT8 transporter (Allan-Herndon-Dudley syndrome) or the thyroid hormone receptor alpha (present in many tissues but not the hypothalamus/pituitary) (31, 32). Here, marked tissue-specific hypothyroidism is seen with dramatic effects on bone and brain development despite “normal” or even raised serum thyroid hormone levels (31, 32). The effects of more common variants are less clear.

At the same time evidence has been accumulating of tissue-specific regulation of thyroid hormone contents in tissues via differential expression of thyroid hormone transporters and iodothyronine deiodinases (29, 33). It has been hypothesized that LT4 monotherapy may not restore intracellular T3 levels in the brain in all patients and this may explain the dissatisfaction some patients have on LT4.

### There Is Dissatisfaction in Individuals With Hypothyroidism Who Are on LT4 Monotherapy

Even with normalization of TSH, patients may still complain of symptoms overlapping with overt hypothyroidism such as lethargy, sleepiness, memory problems, inability to concentrate, and process information (“brain fog”), feeling cold and weight gain (2). Symptoms consistent with the presence of hypothyroidism are very non-specific and cannot be used to differentiate those with hypothyroidism from euthyroid controls (34). Dissatisfaction with LT4 monotherapy is not a new issue. Even in the early 1970's when traditionally higher treatment doses (150 mcg of LT4 and 45 mcg of LT3) were used and treatment initiated at more advanced disease stages, it was recognized that a subset of individuals required LT3 to restore health (35). This would suggest that dissatisfaction is not simply a consequence of persistent symptomatology due to minor thyroid function abnormalities. It is a consistent finding that patients on LT4 replacement even with a normal TSH display significant impairment in psychological well-being compared to controls of similar age and sex (36–39). Whilst this could, at least in part be attributed to disease misclassification (not treating co-existing depression) or confounding it could also indicate that in a proportion of patients there is imperfect replacement with LT4 alone.

For cohort studies and clinical trial design, it is worth considering that standard assessments such as the General Health Questionnaire and Hospital Anxiety and Depression Score may not capture effectively all the symptoms leading to dissatisfaction. Thus, more disease-specific outcome measure such as THYPRO (40) may provide more robust outcome assessment.

### Patients Who Have Their Thyroid Function Tested Are Growing in Number, but Are Not Entirely Representative of the General Population

There has been a steady increase in thyroid function testing in the last two decades (1, 11, 12, 41). In keeping with this, cohort data from Norway has identified that the prevalence of untreated hypothyroidism is 0.1% representing an 84% fall from the 1990s (42). Individuals who have their thyroid function checked are more likely to be female, aged over 60 (43), have higher psychological morbidity (10), but do not appear to have increased rates of hypothyroidism compared to the general population (10). In the UK, the three most common reasons for thyroid function testing that led to a prescription of LT4 are depression, fatigue and weight gain (11). This suggests—perhaps not surprisingly—that individuals with these complaints are preferentially selected for testing for thyroid function and since subclinical hypothyroidism is common (especially in females over 60 where rates approach 10%), a “co-incidental” finding of subclinical hypothyroidism with these symptoms will commonly occur.

Importantly, these potential selection biases will need to be taken into account in cohort studies. Symptoms at initial levothyroxine prescription may need to be considered by selection criteria or minimization depending on the outcome measure used.

### Patient Characteristics Influence Current Prescribing Practice and Treatment Course

Individuals with only borderline abnormalities in TSH are increasingly being started on LT4 (11, 12, 44). It appears that thresholds for levothyroxine initiation differ according to patient symptoms at presentation. For example, individuals with tiredness or depression had levothyroxine initiated at lower TSH thresholds than those who had their thyroid function checked as part of general screening (11). This is pertinent as it is well-established that thyroid-related patient complaints overlap with a plethora of non-specific symptoms which may be caused by other conditions (11, 45, 46). Future trials of combination thyroid hormone replacement need to have inclusion criteria which take into account the patient's thyroid status at initiation of levothyroxine. For instance, individuals with non-specific symptoms and borderline or normal thyroid function (prior to treatment) may have little to gain from either LT4 or combination LT3:LT4 therapy (47, 48).

Being female and having higher net family income are predictive factors for receiving LT4 (49) although it is not clear from this data if this was driven by increased thyroid function testing in these groups. In the UK there is a 49-fold variation in the number of LT3 prescriptions per 1,000 LT4 prescriptions by local prescribing region (19). A substantial driver of this has been the dramatic price rise in LT3 in the UK. Crucially, patients in deprived areas were much less likely to receive LT3, even before the price rises, and this has become more dramatic following prescription cost changes. Cohort studies looking at long term outcomes of LT3 therapy will need to consider social class bias when assessing these outcomes.

Whilst data on the demographics of patients on LT3 are lacking, insights may be attained from studying cohorts of patients on LT4. Individuals treated with LT4 for primary hypothyroidism in the UK were more likely to develop a suppressed TSH following treatment if they originally presented with depression, tiredness or fatigue (11). Women and individuals with a TSH of <4.0 mU/l at index prescription were also more likely to develop a suppressed TSH (11) on LT4 while older individuals and those with cardiovascular risk factors were less likely to (11). It is not unreasonable to suggest that if these symptoms have persisted despite supra-physiological doses of LT4, then these individuals may be more likely to receive a prescription for LT3. This creates two issues, (i) a potential bias in individuals who receive LT3 and (ii) inclusion into LT3 trials of individuals with borderline thyroid function (including top of the normal range) who become classified as hypothyroid because they are receiving LT4. In such individuals LT3 may be of questionable benefit thus negating a treatment response. There is therefore a compelling argument that in the inclusion/exclusion criteria setting, an index TSH threshold before *initial* LT4 prescription should be used; a TSH threshold between 6.0 and 10.0 mU/l at index LT4 prescription may balance the competing priorities of ease of recruitment and ensuring the presence of sufficient thyroid disease.

### Patients on Levothyroxine Replacement Do Not Have “Normal” Thyroid Function

Patients treated with levothyroxine to achieve normal serum TSH values typically have serum triiodothyronine (T3) concentrations in the reference range but with increased levels of T4 resulting in a significantly increased T4/T3 ratio (13, 50). In individuals studied before and after thyroidectomy absolute serum T3 levels change little once TSH is normalized (50, 51), but in individuals with subclinical hypothyroidism where there is increased deiodinase activity and T3 production, treatment with T4 alone results in a fall in absolute T3 serum levels in addition to the rise in T4/T3 levels (52).

Studies of patients established on levothyroxine also reveal that the relationship between TSH and FT3 disassociates on LT4 monotherapy particularly in athyreotic individuals (13, 50, 53). Supra-physiological levels of FT4 are actually required to normalize serum FT3 levels (50). In animals LT4 monotherapy was unable to restore euthyroidism at the tissue levels despite bringing TSH within its reference range (54) and supra-physiological doses were also required to normalize tissue T3 levels (54). In a small number of patients with goiter who received levothyroxine before thyroidectomy, a 33% increase in LT4 dose was required after thyroidectomy to maintain pre-surgical TSH levels (51) but a clear reduction in FT3 was not observed in the same patient before and after thyroidectomy.

It has been reported in cell lines that T4 inhibits the activity and promotes the destruction of the key deiodinase enzyme, D2 that activates T4 to T3 within cells (55). This results in “autoregulation” that serves to protect cells from excess T4, but also to increase local T3 production where T4 levels are reduced. If this mechanism occurs *in vivo*, treating subclinical hypothyroid subjects with T4 alone paradoxically may make their tissue hypothyroidism worse. Note that in the cell line studies, pituitary cells were less prone to such “autoregulation,” suggesting that TSH would still be effectively suppressed with T4 monotherapy despite inhibition of T3 generation within peripheral tissues. As result, paradoxical reduction in local T3 generation on T4 alone would be expected to occur despite normalizing or even suppressing TSH levels. This is likely to be less of a concern in profoundly hypothyroid individuals, in whom any increase in T4 is likely to contribute positively to the generation of intracellular T3 as levels are so low, but result in “net deterioration” in individuals who were initially only subclinically hypothyroid. What is less clear is the extent of other regulators in the thyroid hormone pathway, including FT3 levels and transport. Taken together it is plausible that multiple “hits” are required to induce substantial tissue hypothyroidism, making the percentage of people who will clearly benefit from LT3 difficult to predict.

In support of this hypothesis, a recent large cohort study confirmed that individuals on LT4 had 15–20% lower T3:T4 ratios on LT4 than healthy or matched controls (14). Prolonged exposure to this different ratio appeared to have adverse health outcomes as, LT4 treated individuals had higher a body mass index despite lower calorie consumption, reported less physical activity and were more likely to be taking antidepressants (14), although confounding remains a possibility.

An additional factor is an individually tailored dose of T4 is not widely used, although more mixed doses are being used e.g., 100/125 mcg alternate days. It is also common to encounter a subset of hypothyroid patients who require unexpectedly high doses of T4 or who have erratic control. A variety of factors can cause this including poor compliance, use of different brands, impaired absorption secondary to gastro-intestinal pathology (e.g., coeliac disease), use of other medications (e.g., iron supplements) timing of medication (ingestion of caffeine around the time of T4 will impair absorption) (56, 57). Repeated dose changes and sub-optimal control will also reduce satisfaction with T4. Novel T4 preparations including gel and elixir may improve absorption (58) and may therefore improve satisfaction with LT4 treatment.

### Reliance on TSH Alone May Be Problematic

Due to small changes in thyroid hormone levels resulting in large TSH modifications, TSH has been the preferred diagnostic test for hypothyroidism (6). However, this approach has been challenged (7). Key issues include that its reference-interval is not universally agreed and it is not usually adjusted for other factors such as ethnicity and iodine status. Studies have also identified in athyreotic rats treated with LT4 that the brain, skeletal muscles and liver continue to exhibit markers of hypothyroidism (25). In contrast, in the hypothalamus the effect of T4 on downregulating D2 activity appears to be less as discussed above, which might explain TSH normalization despite tissue hypothyroidism elsewhere. This is important as it is perhaps unlikely therefore that TSH accurately reflects thyroid hormone concentrations in all tissues and organs on T4 alone. By contrast, chronic combination LT3:LT4 therapy was able to normalize thyroid hormone dependent biological parameters in the brain, liver, and skeletal muscle (25).

Restoring TSH to the reference-range has been adopted as the goal of thyroid hormone replacement. However, TSH may not be corrected to an individual's own reference range. Genetic variation in PDE8B is robustly associated with TSH levels (59–61) but this relationship is lost in individuals on LT4, thus indicating that whilst TSH is normalized on LT4 therapy this may not be to an individual's genetic set-point (60). It is well-established that intra-individual variation in thyroid hormone levels is much narrower than inter-individual variation (62) and that thyroid hormone levels in healthy adult individuals are largely genetically determined (59, 61). Thus, individuals could have thyroid function measurements within the normal laboratory reference range which are “abnormal” for them. The clinical implications of this remain unclear, but are pertinent in individuals on thyroid hormone replacement who may attain blood thyroid hormone levels within the reference-range but not at their genetically determined set-point (60). In practice, severe clinical presentations of hypothyroidism have been documented in individuals with only mildly elevated TSH levels (63). Given an individual has a narrow genetically determined set point of thyroid function (62) the long-term health consequences are unclear and merit further study.

### Current LT3 Dosing Strategies Do Not Replicate T3 Levels in Euthyroid Individuals

FT3 levels show a substantial peak 2–4 h after a dose and wear off after 12 h in individuals on a single daily dose of LT3. Similar profiles on FT3 are observed in hypothyroid patients on combination LT3:LT4 therapy (64), LT3 monotherapy (65, 66), and also euthyroid individuals taking LT3 (67). These profiles are markedly different from euthyroid individuals not on thyroid medications. In particular the peak FT3 level is often substantially above the reference-range. Long-acting T3 preparations are in development and may be the key here (68). Long-acting preparations make it easier to monitor dosing, as it possible to assess 24 h T3 exposure from a single measurement. In addition, TSH levels under these state T3 (and T4) conditions are also more likely to be a true reflection of thyroid status compared to the feedback effects of “pulsatile” T3 on TSH levels (66)—see “unknowns” below. Normalization of tissue thyroid status in murine studies was achieved using slow release pellets of T4 and T3 (25). It is worth noting that in contrast to LT3 novel LT4 formulations including gel and elixir are available in most but not all countries (58).

The ratio of LT3:LT4 is also of paramount importance. Given the majority of circulating T3 comes from peripheral conversion of FT4 to FT3, a secretion ratio of FT4:FT3 in healthy individuals is ~14:1 (69, 70). ETA guidelines (26) suggested three possible methods for calculating LT3 and LT4 doses to give a T4:T3 ratio between 13:1 and 20:1 which is close to normal thyroid hormone secretion, however this ratio is generally lower than those used in clinical trials to date (70). Using these recommended ratios (26) a patient on 100 mcg of LT4 would require between 4 and 6 mcg of LT3 which would be impractical to deliver in split doses with current LT3 formulations.

LT3 dosing is also critical when assessing potential benefits. It is worth noting that one of the key early studies which favored LT4 monotherapy in terms of patient preference over LT3:LT4 combination treatment (71) used up to 60 mcg of LT3 a day. Anxiety and palpitations were common at this high dose which may explain why LT4 monotherapy was preferred in this study.

### Combination LT3:LT4 Therapy Has Not Demonstrated Clear Superiority Over LT4 Monotherapy in Clinical Trials

To date there have been at least 13 randomized controlled trials (RCT) comparing efficacy of combination LT4:LT3 therapy vs. LT4 monotherapy (70). Four systematic reviews/meta-analyses (21–24) of these trials of combination LT3:LT4 replacement found no clear benefit over LT4 monotherapy in terms of mood, health-related quality of life, or cognitive function. This data has been taken to suggest that at the population level there is no benefit of using combination therapy over monotherapy. However, in no study to date has physiological replacement been achieved (59). It is noteworthy that Wiersinga et al. (26) reviewed the five cross-over trials (72–76) and identified that despite adequate blinding 48% of all hypothyroid patients preferred the LT3:LT4 combination therapy, 27% preferred LT4 monotherapy, and 25% had no preference. Furthermore, studies in which low or low normal levels of TSH were achieved frequently reported benefit (73, 77–79). Although this has been taken to suggest “overdosing,” the lower TSH levels may relate to the unphysiological nature of the T3 replacement as discussed above. Potentially more robust disease-specific patient reported outcomes such as THYPRO are now available (40). Overall seems reasonable to assume that combination therapy cannot be assumed to have no advantages over monotherapy until a well-designed long-term trial of physiological combination treatment with a sustained release preparation or three times daily dosing of FT3 has been conducted. Such a trial ideally would be adequately powered to assess specific subsets of patients e.g., those with symptomatic disease, established hypothyroidism, and genetic predispositions such as variation in DIO2 (80).

## Unknowns

### Serum TSH May Be Determined Predominantly by Circulating T4, Not T3

While the complex inverse relationship between the thyroid hormones and TSH (6) is well-established, the relative contribution of FT4 and FT3 in regulating TSH levels is less clear. FT4 may have a greater effect on TSH than FT3 (81). As a result, feedback on serum TSH and its production may be predominantly determined by circulating T4, not T3. This becomes highly relevant when the balance between T4 and T3 in the circulation is perturbed by replacement with T4 monotherapy, as is standard in endocrine practice. Thus, TSH levels may appear to be suppressed more easily on LT4 monotherapy. More research is needed to clarify the relative contributions of LT4 and LT3 therapy on TSH levels.

### The Effect of Short Acting LT3 May Have a Disproportionate Effect on TSH

T4 is slowly metabolized with a serum half-life of several days. As a result, once daily dosing results in <20% variation in T3 levels over 24 h. In contrast, T3 has a shorter half-life, and even 2–3 times a day dosing results in substantial fluctuation in T3 levels over 24 h (64). This variation increases with the proportion of T3 in the replacement formulation when combination T3/T4 therapy is used. The effect of this is unclear, but one potential consequence is that spikes of T3 may have a greater suppressive effect on TSH than for the same area under the curve for T3.

### The Role of Circulating T3 Is Unclear

Circulating T3 appears to have a different role in physiology than circulating T4. This is illustrated by several observations, some familiar to endocrinologists and some less familiar. First, even during profound hypothyroidism (e.g., T4 <5.0 pmol/L, TSH > 20 mU/L), T3 is almost always maintained in the reference range. Second, by contrast, during acute illness T3 falls rapidly whereas T4 is maintained, or may even rise (82). Third, 25% of children under 7 years of age have T3 levels above the adult reference range, with a normal TSH (81). Fourth, higher T3 levels in children (and adults) correlate with *increased* body mass index (83). Consistent with this, children with higher T3 levels have earlier onset of puberty (81). Mendelian randomization studies suggest that in fact fat mass somehow causes an increase in T3 levels, rather than vice-versa (83). Taken together, these observations suggest that circulating T3 levels have a role in signaling nutritional or health status to the brain, perhaps more so than being a major source of T3 to tissues.

### The Usefulness of SNPS in Predicting Who Will Benefit From Combination Therapy Is Unclear

Genetic variation in the deiodinases is associated with altered thyroid function and adverse health outcomes (84). However, effects have been modest and not consistently replicated. Individuals with genetic variants in *DIO2* (rs225014) (80) and MCT10 (rs12885300) (77) have shown preference for combination LT3:LT4 therapy with an additive effect (77). However, these studies had limitations due to multiple testing (80) and small sample size (77). Individual SNPs may have poor discriminatory power in determining who might benefit from LT3:LT4 combination therapy. Alternative strategies such as a panel of single nucleotide polymorphisms or the use of thyroid hormone metabolites or metabolomics may provide better insight into tissue thyroid status. Metabolomic profiles are likely to represent a unique method to assess thyroid status as the metabolites released into the serum provide a fingerprint of thyroid hormone action in tissues. Small studies have shown FT4 concentrations are strongly linked to serum acylcarnitines and phosphatidylcholines, indicating enhanced transport of fatty acids to mitochondrion and subsequent β-oxidation (85). More recently a larger study of urinary metabolites revealed positive relations of alanine, trigonelline, and lactic acid with FT4 and negative relations of dimethylamine, glucose, glycine and lactic acid with TSH (86). To date the effect of FT3 on metabolomics has not been studied. This is likely to be an important research area with potential to identify new biomarkers related to thyroid status.

### There Is Limited Data About the Safety Profile of Long-Term LT3 Therapy

An important argument against the widespread use of LT3 is the lack of data on the safety of long-term use. Given the relatively small number of patients using combination LT3:LT4 therapy this is unlikely to be resolved soon. Most of the studies have been of short duration and only one lasted a full year. A recent observational study (87) of 400 patients on LT3 therapy (median duration of therapy 10.9 years) did not identify an increased risk of cardiovascular disease (HR 1.04 95%CI 0.70, 1.54), atrial fibrillation (HR 0.91 95%CI 0.47, 1.75), or fractures (HR 0.79 95%CI 0.49, 1.27), after adjusting for age, despite lower levels of TSH in “ever users” of T3 vs. “never users” (1.07 mU/l vs. 2.08 mU/l, *p* < 0001). An increased risk of anti-psychotic medication prescription was noted in T3 users, which may reflect biases in individuals who receive LT3. It is also worth noting that none of the 13 RCTs comparing combination therapy to LT4 monotherapy showed any increase in adverse events in the combination group, although follow-up was short ranging from 5 to 52 weeks (70).

It would nonetheless be prudent to have agreed monitoring protocols in individuals on long-term LT3 therapy. Sensible assessments would include pulse rate and rhythm, blood pressure, and mood (in particular anxiety). Tests to be considered will include ECG, echocardiograms, and DXA to assess bone density. Having agreed standardized monitoring protocols, ongoing regular assessments in patients on LT3 will be needed to both address and monitor for potential safety concerns.

## Conclusion

It has proved challenging when treating a proportion of patients with hypothyroidism to ensure they are restored to optimal health. This is perhaps surprising considering the high global prevalence of this chronic condition (1). Although current management practices may satisfy the majority of patients, they remain sub-optimal. Key issues affect all stages of management and include excessive thyroid function testing, variation in threshold for treatment by presenting symptoms, widespread over-replacement on LT4 as evidenced by suppressed TSH, and inequality in access to LT3 (19). Using TSH alone to make management decisions may not be effective for at least a sub-group of patients (7). All these factors result in challenging decisions and different priorities for patients and their physicians (88, 89).

Given the importance of thyroid status for health (90) and the substantial dissatisfaction with LT4 replacement (8) further research is urgently needed. There also needs to be studies addressing alternative markers of tissue hypothyroidism other than TSH, such as genetic markers, metabolites, and metabolomic markers. In tandem there needs to be cohort studies of existing patients on combination therapy to identify any potential long-term health complications.

Clinical trials will need to factor in the following to have the best chance of a meaningful result: (i) patient selection to take into account symptoms at first diagnosis, TSH threshold at LT4 initiation, co-morbidities including auto-immune diseases, (ii) Near physiological LT3 therapy in the trials (multi-dosing or long acting LT3) titrated to a physiological T4/T3 ratio and a normal or low normal TSH, (iii) appropriate outcome assessment such as THYPRO, (iv) sufficient power to detect modest effects which would still be of substantial impact at the population given the prevalence of hypothyroidism and dissatisfaction with LT4 therapy, and (v) sufficient power to detect treatment effects in subgroups, such as patients with the DIO2 genotype.

## Author Contributions

PT, CD, and OO drafted and revised the article. VE, IM, and AS revised the article.

### Conflict of Interest

The authors declare that the research was conducted in the absence of any commercial or financial relationships that could be construed as a potential conflict of interest.

## References

[1] Taylor PN Albrecht D Scholz A Gutierrez-Buey G Lazarus JH Dayan CM. Global epidemiology of hyperthyroidism and hypothyroidism. Nat Rev Endocrinol. (2018) 14:301–16. 10.1038/nrendo.2018.18 29569622

[2] Chaker L Bianco AC Jonklaas J Peeters RP. Hypothyroidism. Lancet. (2017) 390:1550–62. 10.1016/S0140-6736(17)30703-1 28336049 PMC6619426

[3] Eligar V Taylor P Okosieme O Leese G Dayan C. Thyroxine replacement: a clinical endocrinologist's viewpoint. Ann Clin Biochem. (2016) 53(Pt 4):421–33. 10.1177/0004563216642255 27126268

[4] McAninch EA Bianco AC. The history and future of treatment of hypothyroidism. Ann Intern Med. (2016) 164:50–6. 10.7326/M15-1799 26747302 PMC4980994

[5] Braverman LE Ingbar SH Sterling K. Conversion of thyroxine (T4) to triiodothyronine (T3) in athyreotic human subjects. J Clin Invest. (1970) 49:855–64. 10.1172/JCI106304 4986007 PMC535757

[6] Hadlow NC Rothacker KM Wardrop R Brown SJ Lim EM Walsh JP The relationship between TSH and free T4 in a large population is complex and non-linear and differs by age and sex. J Clin Endocrinol Metab. (2013) 98:2936–43. 10.1210/jc.2012-4223 23671314

[7] Midgley JEM Toft AD Larisch R Dietrich JW Hoermann R. Time for a reassessment of the treatment of hypothyroidism. BMC Endocr Disord. (2019) 19:37. 10.1186/s12902-019-0365-4 30999905 PMC6471951

[8] Peterson SJ Cappola AR Castro MR Dayan CM Farwell AP Hennessey JV. An online survey of hypothyroid patients demonstrates prominent dissatisfaction. Thyroid. (2018) 28:707–21. 10.1089/thy.2017.0681 29620972 PMC6916129

[9] Wiersinga WM. Paradigm shifts in thyroid hormone replacement therapies for hypothyroidism. Nat Rev Endocrinol. (2014) 10:164–74. 10.1038/nrendo.2013.258 24419358

[10] Bould H Panicker V Kessler D Durant C Lewis G Dayan C. Investigation of thyroid dysfunction is more likely in patients with high psychological morbidity. Fam Pract. (2012) 29:163–7. 10.1093/fampra/cmr059 21890841

[11] Taylor PN Iqbal A Minassian C Sayers A Draman MS Greenwood R. Falling threshold for treatment of borderline elevated thyrotropin levels-balancing benefits and risks: evidence from a large community-based study. JAMA Intern Med. (2014) 174:32–9. 10.1001/jamainternmed.2013.11312 24100714

[12] Medici DBB Nygaard MB Cour DJLL Grand DMK Siersma DV Nicolaisdottir MDR. Changes in prescription routines for treating hypothyroidism between 2001 and 2015—an observational study of 929,684 primary care patients in Copenhagen. Thyroid. (2019) 29. 10.1089/thy.2018.0539 31017048

[13] Gullo D Latina A Frasca F Le Moli R Pellegriti G Vigneri R. Levothyroxine monotherapy cannot guarantee euthyroidism in all athyreotic patients. PLoS ONE. (2011) 6:e22552. 10.1371/journal.pone.0022552 21829633 PMC3148220

[14] Peterson SJ McAninch EA Bianco AC. Is a normal TSH synonymous with “euthyroidism” in levothyroxine monotherapy? J Clin Endocrinol Metab. (2016) 101:4964–73. 10.1210/jc.2016-2660 27700539 PMC6287526

[15] Parle JV Franklyn JA Cross KW Jones SR Sheppard MC Thyroxine prescription in the community: serum thyroid stimulating hormone level assays as an indicator of undertreatment or overtreatment. Br J Gen Pract. (1993) 43:107–9. 8323787 PMC1372330

[16] Okosieme OE Belludi G Spittle K Kadiyala R Richards J. Adequacy of thyroid hormone replacement in a general population. QJM. (2011) 104:395–401. 10.1093/qjmed/hcq222 21109503

[17] Carr D McLeod DT Parry G Thornes HM. Fine adjustment of thyroxine replacement dosage: comparison of the thyrotrophin releasing hormone test using a sensitive thyrotrophin assay with measurement of free thyroid hormones and clinical assessment. Clin Endocrinol. (1988) 28:325–33. 10.1111/j.1365-2265.1988.tb01219.x 3139338

[18] Flynn RW Bonellie SR Jung RT MacDonald TM Morris AD Leese GP. Serum thyroid-stimulating hormone concentration and morbidity from cardiovascular disease and fractures in patients on long-term thyroxine therapy. J Clin Endocrinol Metab. (2010) 95:186–93. 10.1210/jc.2009-1625 19906785

[19] Taylor PN Razvi S Muller I Wass J Dayan CM Chatterjee K. Liothyronine cost and prescriptions in England. Lancet Diab Endocrinol. (2019) 7:11–2. 10.1016/S2213-8587(18)30334-6 30577888

[20] Michaelsson LF Medici BB La Cour JL Selmer C Roder M Perrild H Treating hypothyroidism with thyroxine/triiodothyronine combination therapy in denmark: following guidelines or following trends? Eur Thyroid J. (2015) 4:174–80. 10.1159/000437262 26558234 PMC4637515

[21] Grozinsky-Glasberg S Fraser A Nahshoni E Weizman A Leibovici L. Thyroxine-triiodothyronine combination therapy versus thyroxine monotherapy for clinical hypothyroidism: meta-analysis of randomized controlled trials. J Clin Endocrinol Metab. (2006) 91:2592–9. 10.1210/jc.2006-0448 16670166

[22] Escobar-Morreale HF Botella-Carretero JI Escobar del Rey F Morreale de Escobar G. REVIEW: treatment of hypothyroidism with combinations of levothyroxine plus liothyronine. J Clin Endocrinol Metab. (2005) 90:4946–54. 10.1210/jc.2005-0184 15928247

[23] Joffe RT Marriott M. Thyroid hormone levels and recurrence of major depression. Am J Psychiatry. (2000) 157:1689–91. 10.1176/appi.ajp.157.10.1689 11007728

[24] Ma C Xie J Huang X Wang G Wang Y Wang X Thyroxine alone or thyroxine plus triiodothyronine replacement therapy for hypothyroidism. Nuclear Med Commun. (2009) 30:586–93. 10.1097/MNM.0b013e32832c79e0 19491714

[25] Werneck de Castro JP Fonseca TL Ueta CB McAninch EA Abdalla S Wittmann G. Differences in hypothalamic type 2 deiodinase ubiquitination explain localized sensitivity to thyroxine. J Clin Invest. (2015) 125:769–81. 10.1172/JCI77588 25555216 PMC4319436

[26] Wiersinga WM Duntas L Fadeyev V Nygaard B Vanderpump MP. 2012 ETA guidelines: the use of L-T4 + L-T3 in the treatment of hypothyroidism. Eur Thyroid J. (2012) 1:55–71. 10.1159/000339444 24782999 PMC3821467

[27] Okosieme O Gilbert J Abraham P Boelaert K Dayan C Gurnell M. Management of primary hypothyroidism: statement by the British thyroid association executive committee. Clin Endocrinol. (2016) 84:799–808. 10.1111/cen.12824 26010808

[28] Jonklaas J Bianco AC Bauer AJ Burman KD Cappola AR Celi FS. Guidelines for the treatment of hypothyroidism: prepared by the American thyroid association task force on thyroid hormone replacement. Thyroid. (2014) 24:1670–751. 10.1089/thy.2014.0028 25266247 PMC4267409

[29] Bianco AC Casula S. Thyroid hormone replacement therapy: three ‘simple' questions, complex answers. Eur Thyroid J. (2012) 1:88–98. 10.1159/000339447 24783002 PMC3821470

[30] Arrojo e Drigo R Fonseca TL Werneck-de-Castro JPS Bianco AC. Role of the type 2 iodothyronine deiodinase (D2) in the control of thyroid hormone signaling. Biochim Biophys Acta. (2013) 1830:3956–64. 10.1016/j.bbagen.2012.08.019 22967761 PMC4979226

[31] Bochukova E Schoenmakers N Agostini M Schoenmakers E Rajanayagam O Keogh JM. A mutation in the thyroid hormone receptor alpha gene. N Engl J Med. (2012) 366:243–9. 10.1056/NEJMoa1110296 22168587

[32] Schwartz CE May MM Carpenter NJ Rogers RC Martin J Bialer MG. Allan-Herndon-Dudley syndrome and the monocarboxylate transporter 8 (MCT8) gene. Am J Hum Genet. (2005) 77:41–53. 10.1086/431313 15889350 PMC1226193

[33] Escobar-Morreale HF Obregon MJ Escobar del Rey F Morreale de Escobar G. Tissue-specific patterns of changes in 3,5,3′-triiodo-L-thyronine concentrations in thyroidectomized rats infused with increasing doses of the hormone. Which are the regulatory mechanisms? Biochimie. (1999) 81:453–62. 10.1016/S0300-9084(99)80095-9 10403175

[34] Canaris GJ Steiner JF Ridgway EC. Do traditional symptoms of hypothyroidism correlate with biochemical disease? J Gen Intern Med. (1997) 12:544–50. 10.1046/j.1525-1497.1997.07109.x 9294788 PMC1497160

[35] Taylor S Kapur M Adie R. Combined thyroxine and triiodothyronine for thyroid replacement therapy. Br Med J. (1970) 2:270–1. 10.1136/bmj.2.5704.270 5420176 PMC1700438

[36] Saravanan P Visser TJ Dayan CM Psychological well-being correlates with free thyroxine but not free 3,5,3′-triiodothyronine levels in patients on thyroid hormone replacement. J Clin Endocrinol Metab. (2006) 91:3389–93. 10.1210/jc.2006-0414 16804044

[37] Saravanan P Chau WF Roberts N Vedhara K Greenwood R Dayan CM. Psychological well-being in patients on 'adequate' doses of l-thyroxine: results of a large, controlled community-based questionnaire study. Clin Endocrinol. (2002) 57:577–85. 10.1046/j.1365-2265.2002.01654.x 12390330

[38] Panicker V Evans J Bjoro T Asvold BO Dayan CM Bjerkeset O A paradoxical difference in relationship between anxiety, depression and thyroid function in subjects on and not on T4: findings from the HUNT study. Clin Endocrinol. (2009) 71:574–80. 10.1111/j.1365-2265.2008.03521.x 19751298

[39] Wekking EM Appelhof BC Fliers E Schene AH Huyser J Tijssen JG. Cognitive functioning and well-being in euthyroid patients on thyroxine replacement therapy for primary hypothyroidism. Eur J Endocrinol. (2005) 153:747–53. 10.1530/eje.1.02025 16322379

[40] Watt T Cramon P Hegedus L Bjorner JB Bonnema SJ Rasmussen AK. The thyroid-related quality of life measure ThyPRO has good responsiveness and ability to detect relevant treatment effects. J Clin Endocrinol Metab. (2014) 99:3708–17. 10.1210/jc.2014-1322 25004246

[41] Meyerovitch J Rotman-Pikielny P Sherf M Battat E Levy Y Surks MI. Serum thyrotropin measurements in the community: five-year follow-up in a large network of primary care physicians. Arch Intern Med. (2007) 167:1533–8. 10.1001/archinte.167.14.1533 17646608

[42] Asvold BO Vatten LJ Bjoro T. Changes in the prevalence of hypothyroidism: the HUNT Study in Norway. Eur J Endocrinol. (2013) 169:613–20. 10.1530/EJE-13-0459 23975540

[43] Werhun A Hamilton W. Thyroid function testing in primary care: overused and under-evidenced? A study examining which clinical features correspond to an abnormal thyroid function result. Family Practice. (2015) 32:187–91. 10.1093/fampra/cmv010 25782692

[44] Leese GP Flynn RV Jung RT Macdonald TM Murphy MJ Morris AD. Increasing prevalence and incidence of thyroid disease in Tayside, Scotland: the Thyroid Epidemiology Audit and Research Study (TEARS). Clin Endocrinol. (2008) 68:311–6. 10.1111/j.1365-2265.2007.03051.x 17970771

[45] Canaris GJ Manowitz NR Mayor G Ridgway EC. The Colorado thyroid disease prevalence study. Arch Intern Med. (2000) 160:526–34. 10.1001/archinte.160.4.526 10695693

[46] Massolt ET van der Windt M Korevaar TIM Kam BLR Burger JW Franssen GJH. Thyroid hormone and its metabolites in relation to quality of life in patients treated for differentiated thyroid cancer. Clin Endocrinol. (2016) 85:781–8. 10.1111/cen.13101 27175823

[47] Stott DJ Rodondi N Kearney PM Ford I Westendorp RGJ Mooijaart SP. Thyroid hormone therapy for older adults with subclinical hypothyroidism. N Engl J Med. (2017) 376:2534–44. 10.1056/NEJMoa1603825 28402245

[48] Pollock MA Sturrock A Marshall K Davidson KM Kelly CJ McMahon AD. Thyroxine treatment in patients with symptoms of hypothyroidism but thyroid function tests within the reference range: randomised double blind placebo controlled crossover trial. BMJ. (2001) 323:891–5. 10.1136/bmj.323.7318.891 11668132 PMC58535

[49] Olmos RD Figueiredo RCD Aquino EM Lotufo PA Bensenor IM. Gender, race and socioeconomic influence on diagnosis and treatment of thyroid disorders in the Brazilian Longitudinal Study of Adult Health (ELSA-Brasil). Brazil J Med Biol Res. (2015) 48:751–8. 10.1590/1414-431x20154445 26108100 PMC4541696

[50] Ito M Miyauchi A Morita S Kudo T Nishihara E Kihara M. TSH-suppressive doses of levothyroxine are required to achieve preoperative native serum triiodothyronine levels in patients who have undergone total thyroidectomy. Eur J Endocrinol. (2012) 167:373–8. 10.1530/EJE-11-1029 22711760

[51] Del Duca SC Santaguida MG Brusca N Gatto I Cellini M Gargano L. Individually-tailored thyroxine requirement in the same patients before and after thyroidectomy: a longitudinal study. Eur J Endocrinol. (2015) 173:351–7. 10.1530/EJE-15-0314 26092761

[52] Meier C Staub JJ Roth CB Guglielmetti M Kunz M Miserez AR. TSH-controlled L-thyroxine therapy reduces cholesterol levels and clinical symptoms in subclinical hypothyroidism: a double blind, placebo-controlled trial (Basel Thyroid Study). J Clin Endocrinol Metab. (2001) 86:4860–6. 10.1210/jcem.86.10.7973 11600554

[53] Hoermann R Midgley JEM Giacobino A Eckl WA Wahl HG Dietrich JW. Homeostatic equilibria between free thyroid hormones and pituitary thyrotropin are modulated by various influences including age, body mass index and treatment. Clin Endocrinol. (2014) 81:907–15. 10.1111/cen.12527 24953754

[54] Escobar-Morreale HF Obregon MJ Escobar del Rey F Morreale de Escobar G Replacement therapy for hypothyroidism with thyroxine alone does not ensure euthyroidism in all tissues, as studied in thyroidectomized rats. J Clin Invest. (1995) 96:2828–38. 10.1172/JCI118353 8675653 PMC185993

[55] Christoffolete MA Ribeiro R Singru P Fekete C da Silva WS Gordon DF. Atypical expression of type 2 iodothyronine deiodinase in thyrotrophs explains the thyroxine-mediated pituitary thyrotropin feedback mechanism. Endocrinology. (2006) 147:1735–43. 10.1210/en.2005-1300 16396983

[56] Centanni M Benvenga S Sachmechi I. Diagnosis and management of treatment-refractory hypothyroidism: an expert consensus report. J Endocrinol Invest. (2017) 40:1289–301. 10.1007/s40618-017-0706-y 28695483 PMC5680379

[57] Virili C Antonelli A Santaguida MG Benvenga S Centanni M. Gastrointestinal malabsorption of thyroxine. Endocr Rev. (2019) 40:118–36. 10.1210/er.2018-00168 30476027

[58] Vita R Fallahi P Antonelli A Benvenga S The administration of L-thyroxine as soft gel capsule or liquid solution. Expert Opin Drug Deliv. (2014) 11:1103–11. 10.1517/17425247.2014.918101 24896369

[59] Taylor PN Porcu E Chew S Campbell PJ Traglia M Brown SJ Whole-genome sequence-based analysis of thyroid function. Nat Commun. (2015) 6:5681 10.1038/ncomms6681 25743335 PMC4366514

[60] Taylor PN Panicker V Sayers A Shields B Iqbal A Bremner AP. A meta-analysis of the associations between common variation in the PDE8B gene and thyroid hormone parameters, including assessment of longitudinal stability of associations over time and effect of thyroid hormone replacement. Eur J Endocrinol. (2011) 164:773–80. 10.1530/EJE-10-0938 21317282 PMC3080745

[61] Teumer A Chaker L Groeneweg S Li Y Di Munno C Barbieri C. Genome-wide analyses identify a role for SLC17A4 and AADAT in thyroid hormone regulation. Nat Commun. (2018) 9:4455. 10.1038/s41467-018-06356-1 30367059 PMC6203810

[62] Andersen S Pedersen KM Bruun NH Laurberg P. Narrow individual variations in serum T(4) and T(3) in normal subjects: a clue to the understanding of subclinical thyroid disease. J Clin Endocrinol Metab. (2002) 87:1068–72. 10.1210/jcem.87.3.8165 11889165

[63] Mallipedhi A Vali H Okosieme O. Myxedema coma in a patient with subclinical hypothyroidism. Thyroid. (2011) 21:87–9. 10.1089/thy.2010.0175 21058937

[64] Saravanan P Siddique H Simmons DJ Greenwood R Dayan CM Twenty-four hour hormone profiles of TSH, Free T3 and free T4 in hypothyroid patients on combined T3/T4 therapy. Exp Clin Endocrinol Diab. (2007) 115:261–7. 10.1055/s-2007-973071 17479444

[65] Saberi M Utiger RD. Serum thyroid hormone and thyrotropin concentrations during thyroxine and triiodothyronine therapy. J Clin Endocrinol Metab. (1974) 39:923–7. 10.1210/jcem-39-5-923 4422006

[66] Jonklaas J Burman KD. Daily administration of short-acting liothyronine is associated with significant triiodothyronine excursions and fails to alter thyroid-responsive parameters. Thyroid. (2016) 26:770–8. 10.1089/thy.2015.0629 27030088 PMC4913511

[67] Leggio GM Incognito T Privitera G Marano MR Drago F. Comparative bioavailability of different formulations of levothyroxine and liothyronine in healthy volunteers. J Endocrinol Invest. (2006) 29:Rc35–38. 10.1007/BF03349205 17259789

[68] Da Conceicao RR Fernandes GW Fonseca TL Bocco B Bianco AC. Metal coordinated poly-zinc-liothyronine provides stable circulating triiodothyronine levels in hypothyroid rats. Thyroid. (2018) 28:1425–33. 10.1089/thy.2018.0205 30301431 PMC7207055

[69] Pilo A Iervasi G Vitek F Ferdeghini M Cazzuola F Bianchi R. Thyroidal and peripheral production of 3,5,3′-triiodothyronine in humans by multicompartmental analysis. Am J Physiol. (1990) 258:E715–26. 10.1152/ajpendo.1990.258.4.E715 2333963

[70] Dayan C Panicker V. Management of hypothyroidism with combination thyroxine (T4) and triiodothyronine (T3) hormone replacement in clinical practice: a review of suggested guidance. Thyroid Res. (2018) 11:1. 10.1186/s13044-018-0045-x 29375671 PMC5772692

[71] Smith RN Taylor SA Massey JC. Controlled clinical trial of combined triiodothyronine and thyroxine in the treatment of hypothyroidism. Br Med J. (1970) 4:145–8. 10.1136/bmj.4.5728.145 4097650 PMC1819870

[72] Walsh JP Shiels L Lim EM Bhagat CI Ward LC Stuckey BG. Combined thyroxine/liothyronine treatment does not improve well-being, quality of life, or cognitive function compared to thyroxine alone: a randomized controlled trial in patients with primary hypothyroidism. J Clin Endocrinol Metab. (2003) 88:4543–50. 10.1210/jc.2003-030249 14557419

[73] Nygaard B Jensen EW Kvetny J Jarlov A Faber J. Effect of combination therapy with thyroxine (T4) and 3,5,3′-triiodothyronine versus T4 monotherapy in patients with hypothyroidism, a double-blind, randomised cross-over study. Eur J Endocrinol. (2009) 161:895–902. 10.1530/EJE-09-0542 19666698

[74] Bunevicius R Kazanavicius G Zalinkevicius R Prange AJ Jr. Effects of thyroxine as compared with thyroxine plus triiodothyronine in patients with hypothyroidism. N Engl J Med. (1999) 340:424–9. 10.1056/NEJM199902113400603 9971866

[75] Escobar-Morreale HF Botella-Carretero JI Gomez-Bueno M Galan JM Barrios V Sancho J. Thyroid hormone replacement therapy in primary hypothyroidism: a randomized trial comparing L-thyroxine plus liothyronine with L-thyroxine alone. Ann Intern Med. (2005) 142:412–24. 10.7326/0003-4819-142-6-200503150-00007 15767619

[76] Bunevicius R Jakubonien N Jurkevicius R Cernicat J Lasas L Prange AJ Jr. Thyroxine vs. thyroxine plus triiodothyronine in treatment of hypothyroidism after thyroidectomy for Graves' disease. Endocrine. (2002) 18:129–33. 10.1385/ENDO:18:2:129 12374459

[77] Carle A Faber J Steffensen R Laurberg P Nygaard B Hypothyroid patients encoding combined MCT10 and DIO2 gene polymorphisms may prefer L-T3 + L-T4 combination treatment - data using a blind, randomized, clinical study. Eur Thyroid J. (2017) 6:143–51. 10.1159/000469709 28785541 PMC5527224

[78] Appelhof BC Fliers E Wekking EM Schene AH Huyser J Tijssen JG Combined therapy with levothyroxine and liothyronine in two ratios, compared with levothyroxine monotherapy in primary hypothyroidism: a double-blind, randomized, controlled clinical trial. J Clin Endocrinol Metab. (2005) 90:2666–74. 10.1210/jc.2004-2111 15705921

[79] Hoang TD Olsen CH Mai VQ Clyde PW Shakir MK Desiccated thyroid extract compared with levothyroxine in the treatment of hypothyroidism: a randomized, double-blind, crossover study. J Clin Endocrinol Metab. (2013) 98:1982–90. 10.1210/jc.2012-4107 23539727

[80] Panicker V Saravanan P Vaidya B Evans J Hattersley AT Frayling TM Common variation in the DIO2 gene predicts baseline psychological well-being and response to combination thyroxine plus triiodothyronine therapy in hypothyroid patients. J Clin Endocrinol Metab. (2009) 94:1623–9. 10.1210/jc.2008-1301 19190113

[81] Taylor PN Sayers A Okosieme O Das G Draman MS Tabasum A. Maturation in serum thyroid function parameters over childhood and puberty: results of a longitudinal study. J Clin Endocrinol Metab. (2017) 102:2508–15. 10.1210/jc.2016-3605 28472343 PMC5505201

[82] Peeters RP Wouters PJ van Toor H Kaptein E Visser TJ Van den Berghe G. Serum 3,3′,5′-triiodothyronine (rT3) and 3,5,3′-triiodothyronine/rT3 are prognostic markers in critically ill patients and are associated with postmortem tissue deiodinase activities. J Clin Endocrinol Metab. (2005) 90:4559–65. 10.1210/jc.2005-0535 15886232

[83] Taylor PN Richmond R Davies N Sayers A Stevenson K Woltersdorf W. Paradoxical relationship between body mass index and thyroid hormone levels: a study using mendelian randomization. J Clin Endocrinol Metab. (2016) 101:730–8. 10.1210/jc.2015-3505 26595101 PMC4880123

[84] Taylor PN Peeters R Dayan CM. Genetic abnormalities in thyroid hormone deiodinases. Curr Opin Endocrinol Diabet Obes. (2015) 22:402–6. 10.1097/MED.0000000000000180 26192703

[85] Jourdan C Linseisen J Meisinger C Petersen AK Gieger C Rawal R Associations between thyroid hormones and serum metabolite profiles in an euthyroid population. Metabolomics. (2014) 10:152–64. 10.1007/s11306-013-0563-4 24955082 PMC4042025

[86] Friedrich N Pietzner M Cannet C Thuesen BH Hansen T Wallaschofski H Urinary metabolomics reveals glycemic and coffee associated signatures of thyroid function in two population-based cohorts. PLoS ONE. (2017) 12:e0173078 10.1371/journal.pone.0173078 28253303 PMC5333857

[87] Leese GP Soto-Pedre E Donnelly LA. Liothyronine use in a 17 year observational population-based study - the tears study. Clin Endocrinol. (2016) 85:918–25. 10.1111/cen.13052 26940864

[88] Dew R King K Okosieme OE Pearce SH Donovan G Taylor PN. Attitudes and perceptions of health professionals towards management of hypothyroidism in general practice: a qualitative interview study. BMJ open. (2018) 8:e019970. 10.1136/bmjopen-2017-019970 29467136 PMC5855452

[89] Dew R King K Okosieme OE Pearce S Donovan G Taylor P. Patients' attitudes and perceptions towards treatment of hypothyroidism in general practice: an in-depth qualitative interview study. BJGP open. (2017) 1:bjgpopen17X100977. 10.3399/bjgpopen17X100977 30564669 PMC6169953

[90] Taylor PN Razvi S Pearce SH Dayan CM. Clinical review: a review of the clinical consequences of variation in thyroid function within the reference range. J Clin Endocrinol Metab. (2013) 98:3562–71. 10.1210/jc.2013-1315 23824418

